# Structural definition and substrate specificity of the S28 protease family: the crystal structure of human prolylcarboxypeptidase

**DOI:** 10.1186/1472-6807-10-16

**Published:** 2010-06-11

**Authors:** Stephen M Soisson, Sangita B Patel, Pravien D Abeywickrema, Noel J Byrne, Ronald E Diehl, Dawn L Hall, Rachael E Ford, John C Reid, Keith W Rickert, Jennifer M Shipman, Sujata Sharma, Kevin J Lumb

**Affiliations:** 1 Global Structural Biology, Merck Research Laboratories, P.O. Box 4, West Point, PA 19486, USA

## Abstract

**Background:**

The unique S28 family of proteases is comprised of the carboxypeptidase PRCP and the aminopeptidase DPP7. The structural basis of the different substrate specificities of the two enzymes is not understood nor has the structure of the S28 fold been described.

**Results:**

The experimentally phased 2.8 Å crystal structure is presented for human PRCP. PRCP contains an α/β hydrolase domain harboring the catalytic Asp-His-Ser triad and a novel helical structural domain that caps the active site. Structural comparisons with prolylendopeptidase and DPP4 identify the S1 proline binding site of PRCP. A structure-based alignment with the previously undescribed structure of DPP7 illuminates the mechanism of orthogonal substrate specificity of PRCP and DPP7. PRCP has an extended active-site cleft that can accommodate proline substrates with multiple N-terminal residues. In contrast, the substrate binding groove of DPP7 is occluded by a short amino-acid insertion unique to DPP7 that creates a truncated active site selective for dipeptidyl proteolysis of N-terminal substrates.

**Conclusion:**

The results define the structure of the S28 family of proteases, provide the structural basis of PRCP and DPP7 substrate specificity and enable the rational design of selective PRCP modulators.

## Background

Proteases are an important class of enzymes involved in a diverse range of physiological processes. The modulation of proteolytic activity is an established means of therapeutic intervention with currently marketed products for afflictions as diverse as type 2 diabetes, hypertension and viral infections. The human protease tree is comprised of at least 676 diverse proteins that have been systematically organized into clans and families based on similarity in sequence, structure, and function [1]. Although the structural basis of catalytic mechanism, substrate specificity and rational drug design has been identified for numerous protease families, there has been no structural description of the S28 family of proteases that form a distinct branch of the serine carboxypeptidase clan.

The S28 family of peptidases consists of two enzymes, PRCP and DPP7. DPP7 is also called dipeptidyl peptidase 2 and quiescent cell proline dipeptidase [2-4]. PRCP is a lysosomal, serine carboxypeptidase that cleaves hydrophobic C-terminal amino acids adjacent to proline [5,6]. In contrast, DPP7 is a serine dipeptidyl aminopeptidase that cleaves N-terminal amino acids adjacent to proline and is localized to intracellular vesicles [3].

Human PRCP and human DPP7 share 39.6% sequence identity and 55.4% sequence similarity. At the sequence level, the two enzymes are unrelated to other proteases; the next closest human homologues are PEP (8.4% sequence identity and 13.9% sequence similarity) and DPP4 (6.5% sequence identity and 11.2% sequence similarity). The S28 proteases PRCP and DPP7 are therefore unique within the protease superfamily.

PRCP was originally discovered as an angiotensinase [7] and has since been implicated in vasodilatory, proinflammatory, and metabolic pathways [6,8,9]. For example, angiotensin II, III and prekallikrein are all inactivated by PRCP, implicating a role for the enzyme in hypertension, tissue proliferation and smooth muscle growth. PRCP is also reported to inactivate α-melanocyte-stimulating hormone, a neuropeptide that plays a role in regulating appetite [10]. DPP7 has been implicated in apoptosis in quiescent lymphocytes [3].

Here we report the crystal structure of human PRCP. The enzyme consists of an α/β hydrolase domain that contains a unique structural domain insertion that caps the active site. Comparison with the recently released coordinates of DPP7 illuminates the structural basis for the different substrate specificities of PRCP and DPP7. The results lay the foundation for understanding the structural basis of PRCP activity and for the structure-guided discovery of PRCP modulators for target validation and disease modification.

## Results and Discussion

### The structure of PRCP

The crystal structure of human PRCP was determined using MIRAS phasing techniques at 2.8 Å resolution by analyzing native, mercury and platinum-derivatized crystals (Table 1). Interpretation of heavy-atom positions and resultant electron-density maps show that the asymmetric unit contains one molecule of PRCP with an unusually high solvent content of 82%. Although the derivative data sets provided modest amounts of phase information, a very high-quality experimental electron density map was obtained. These results reflect the high redundancy of the data and high solvent content of the crystals.

**Table 1:** Structure determination statistics.

	Native	EMTS	K_2_PtCl_4_
Resolution (Å)	2.80	2.8	3.0
Space group	R32	R32	R32
Unit-cell dimensions (Å)	*a *= *b *= 181.14 *c *= 240.13	*a *= *b *= 179.76 *c *= 240.90	*a *= *b *= 181.27 *c *= 240.02
Completeness (%)	100 (100)	99.9 (100)	95.8 (69.6)
Redundancy	10.9	10.2	5.1
*R*_sym _(%)	9.8 (59.4)	8.4 (60.9)	11.5 (38.3)
*R_merge_*(%)	-	24.2	18.3
Heavy atom sites	-	20	13
Isomorphous phasing power	-	1.25/1.22	1.28/1.36
Anomalous phasing power	-	0.482	0.335
Isomorphous R*_cullis_*	-	0.784/0.722	0.717/0.595
Anomalous R*_cullis_*	-	0.656	0.947
Overall FOM	0.344/0.426		
Resolution (Å)	50.0-2.79		
*R_f _*(%)	21.8		
*R_free _*(%)	24.1		
Protein atoms	3529		
Water molecules	93		

Values in parentheses are for the highest resolution bin.

The experimental maps allowed nearly the entire structure of PRCP to be modeled and subsequently refined. The final refined model consists of residues 46-348 and 353-491, five N-linked glycans, and four disulfide bridges (residues 215-372, 233-310, 264-343 and 364-394). The final R and R_free _values are 21.8% and 24.1%, respectively. Geometry and stereochemistry are good with 95% of the residues in the most favored region of the Ramachandran plot and an overall MolProbity score of 88%. One region of unexplained tubular electron density is observed in the S1' active site area that may correspond to a structurally heterogeneous population of bound polymer (e.g., polyethylene glycol) and a second peak of unexplained electron density is observed near the putative proline S1 binding site.

**Figure 1:**
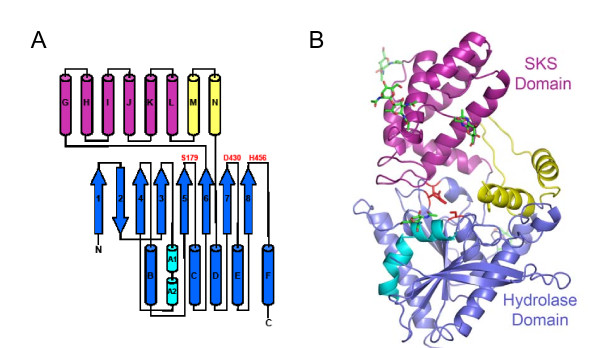
**The structure of PRCP**. (A) Schematic diagram of the secondary structure of PRCP. The canonical α/β hydrolase fold is shown in blue and cyan, the SKS domain in magenta, and the M and N helices of the hydrolase insert domain in yellow. (B) Ribbon diagram of tertiary structure of PRCP. The Asp-His-Ser catalytic triad is shown in red. Glycans are shown in green.

The overall architecture of PRCP consists of two main structural entities: an α/β hydrolase domain and a novel SKS domain (Figures 1A and 1B). The α/β hydrolase domain is constructed from two non-contiguous stretches of PRCP (residues 46-204 and 405-491). Although a number of β-sheet topologies have been described for the α/β hydrolase fold [11], the β-sheet topology of PRCP is identical to the prototypical α/β hydrolase fold [12].

The unique insertion in the PRCP hydrolase domain occurs between strand 6 and helix D and spans residues 194-398. The first part of the insertion (residues 194-334) consists of five helices packed into a novel helical bundle (the SKS domain) that caps the active site (Figure 1B). A DALI search to identify structures containing similar helical bundles to the SKS domain did not identify proteins with similar folds (Z scores < 3.3), suggesting that the SKS domain is a novel structural motif.

Four residues following the SKS domain are likely disordered as evidenced by a lack of electron density (residues 349-352). The region is followed by a pair of helices (M and N) that are linked by two long, irregular, loosely packed strands that form a concave surface at one entrance to the active site. The irregular strands and the M and N helices appear to provide additional stabilizing interactions between the SKS and hydrolase domains and form part of the substrate binding surface (Figure 1B).

Previously reported mass-spectrometry results are consistent with the CHO-expressed PRCP protein containing about 9 kDa of glycan [13]. Sequence analysis suggests that there are six possible N-glycosylation sites at asparagines 47, 101, 317, 336, 345 and 415 that correspond to the canonical glycosylation sequence Asn-Xaa-Ser/Thr [14]. Asn 47 is not glycosylated in the structure, in accord with mass-spectrometric mapping of glycan sites [13]. Clear evidence of covalently attached and ordered saccharide is observed at the other five canonical glycosylation sites (Figure 1B). The presence of extensive glycosylation is likely a contributing factor to the high solvent content of the crystals.

**Figure 2:**
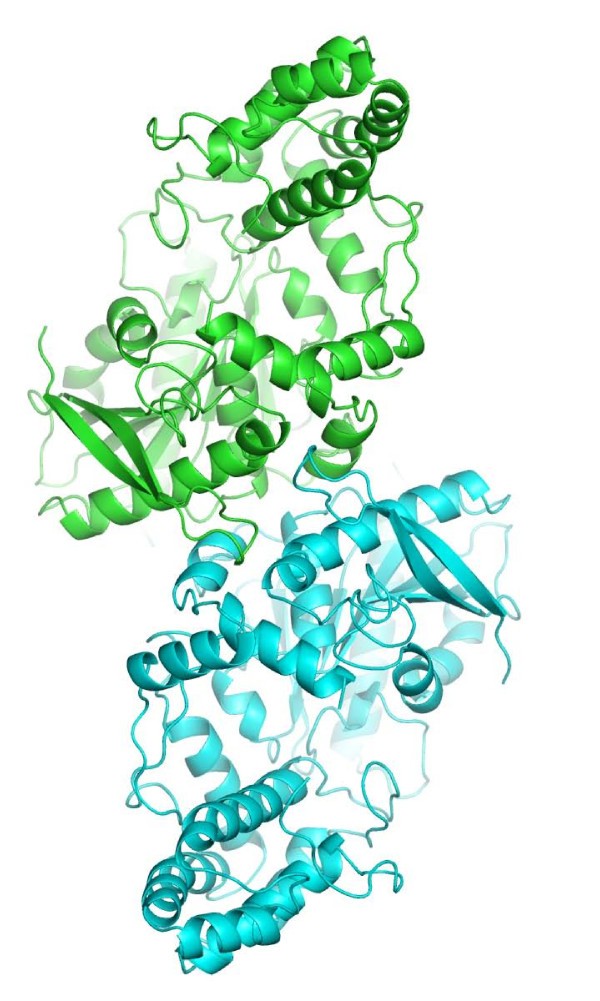
**The PRCP Dimer**. The crystallographic dimer of PRCP with monomer subunits shown in green and blue.

A crystallographic dimerization interface is seen that involves the hydrolase domain (Figure 2). The dimer interface is formed through packing interactions across a two-fold crystallographic symmetry axis present in the crystal, and buries approximately 3600 Å^2 ^of surface area on the combined molecules of PRCP. This observation is consistent with previous gel-filtration and dynamic-light scattering results suggesting that PRCP is a dimer in solution [6,13,15].

### The active site of PRCP

**Figure 3:**
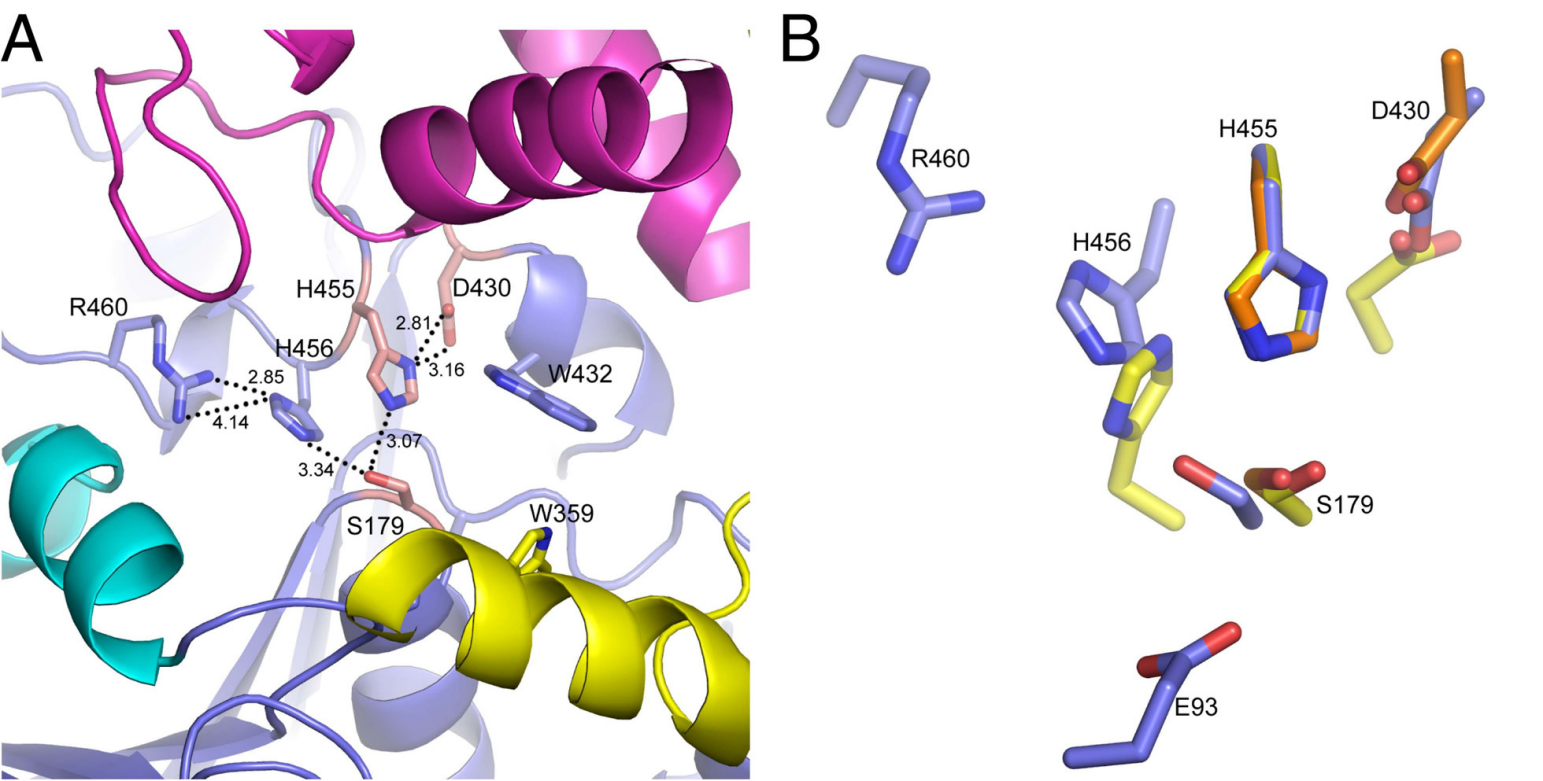
**The active site of PRCP**. (A) The active site of PRCP. The Asp-His-Ser catalytic triad is shown in salmon. Additional active site side chain features discussed in the text are colored according to structural element as in Figure 1. (B) Structural alignment of PRCP active site (blue), DPP4 (orange), and human pancreatic lipase (yellow) aligned on the catalytic histidine. PRCP residues are labeled.

The catalytic triad consisting of Ser 179, Asp 430 and His 455 (Figure 3A) can be identified based on structural similarity with other hydrolases [12]. Ser 179 is located on a sharp turn between strand 5 and helix C, Asp 430 is located between strand 7 and helix E, and His 455 is located on the loop between strand 8 and helix F. Ser 179, Asp 430 and His 455 are spatially oriented in the same catalytic triad arrangement seen in other serine α/β hydrolases such as DPP4 (Figure 3B). The active site is located on one face of the hydrolase domain and is capped by the SKS domain. The putative oxyanion hole is formed by the backbone amide nitrogen atoms of Tyr 180 and Gly 181. The side chain of Asn 92 is also positioned in close proximity to the putative oxyanion hole and may play a role in stabilizing the negatively charged tetrahedral intermediate formed during catalysis. Other proteases containing the hydrolase fold adopt a similar arrangement of the catalytic triad and oxyanion hole, although the nature of the capping domain varies. For example, DPP4 and PEP are capped with a β-propeller domain [16-18] and human protective protein is capped with a helical bundle unrelated to the SKS domain [19].

An unanticipated feature of the PRCP active site is an apparent charge-relay system that links the catalytic histidine (His 455) with His 456 and Arg 460 (Figure 3A). The arrangement of side chains places the imidazole nitrogen atoms of His 455 and His 456 within 3.0-3.5 Å of the catalytic serine. The guanidinium group of Arg 460 is in hydrogen bond distance (2.8 Å) of the imidazole ring of His 456. It seems likely that this unique arrangement of residues plays a role in the catalytic mechanism of PRCP. Furthermore, it is possible that the presence of the formally charged Arg 460 in close contact with the tandem histidines could alter the pK_a _of His 455 contributing to the acidic pH optimum (5.5) for both PRCP and DPP7 [6,20,21].

The tandem His-His arrangement is not seen in other serine α/β hydrolases with the exception of the lipases. For example, pancreatic lipase [22], contains a second histidine residue located spatially adjacent to the catalytic histidine in the active site (Figure 3B). In the lipases, the equivalent second His residue is contributed to the active site by a different structural element of the α/β hydrolase fold, and may therefore represent a convergent evolution of the S28 protease family and the lipases. The structural conservation underscores the potential importance of the histidine pair in catalysis.

### Recognition of Pro-X peptide substrates by PRCP

PRCP cleaves carboxy-terminal residues of peptide substrates that contain a penultimate proline. This is exemplified by angiotensin II, the first substrate identified for PRCP (NDRVYIH**P**F) [23] and bradykinin (RPPGFS**P**F) [8]. In contrast, peptides lacking the penultimate Pro, such as angiotensin I (DRVYIH**P**FHL), are not substrates for the enzyme [21].

**Figure 4:**
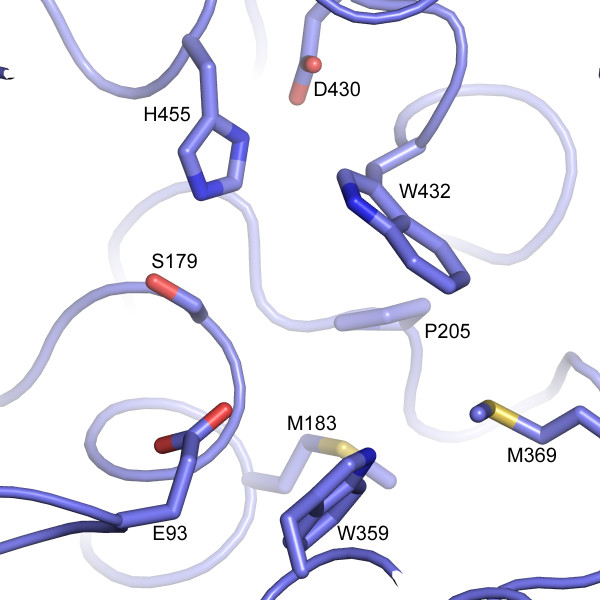
**The hydrophobic substrate-binding pocket of PRCP**. The putative proline-binding pocket is formed by Trp 432 and Trp 359 based on structural identity with other prolyl peptidases and the spatial alignment with the Ser 179-His 455-Asp 430 catalytic triad.

**Figure 5:**
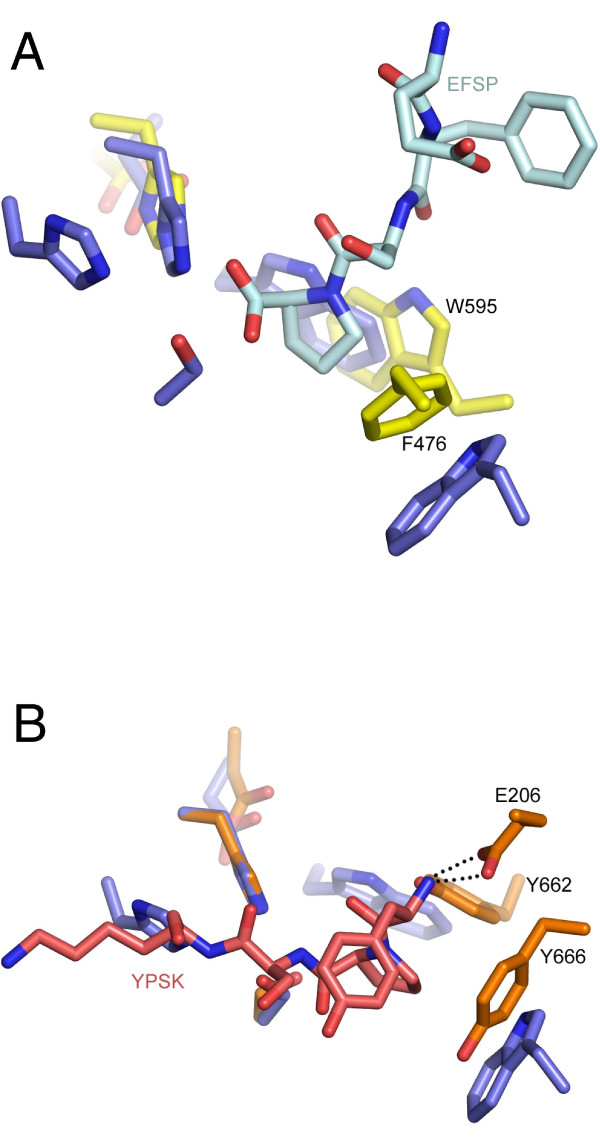
**Identification of the proline recognition site**. (A) Structural alignment of PRCP (blue) with porcine PEP (yellow) in complex with the peptide substrate EFSP (PDB code: 1UOQ) in light blue. Residues discussed in the text are shown as sticks and labeled. (B) Structural alignment of PRCP (blue) with human DPP4 (orange) in complex with the peptide substrate YPSK (PDB code: 1R9N) in pink.

Examination of the active site for potential substrate recognition pockets reveals the presence of a hydrophobic pocket adjacent to the catalytic serine. This pocket is formed primarily by Met 183, Met 369, Trp 432 and Trp 459 (Figure 4). The proline S1 binding site of other proline peptidases is illustrated by PEP and DPP4, which share limited sequence similarity to PRCP and to each other (8% over the hydrolase domain). The structure of substrate-bound PEP [24] shows that the Pro residue of the cleaved substrate (EFSP) is located in a hydrophobic pocket formed by Trp 595 and Phe 476 that is adjacent to the catalytic serine (Figure 5A). Similarly, the structure of DPP4 with a bound peptide substrate shows that the substrate proline is recognized in a pocket formed by Tyr 662 and Tyr 666 (Figure 5B). Structural alignments strongly suggest that the hydrophobic pocket in PRCP, formed by Trp 432, Trp 459, Met 183 and Met 369 is functionally equivalent to the S1 binding sites of PEP and DPP4.

### Substrate specificity of the S28 protease family

**Figure 6:**
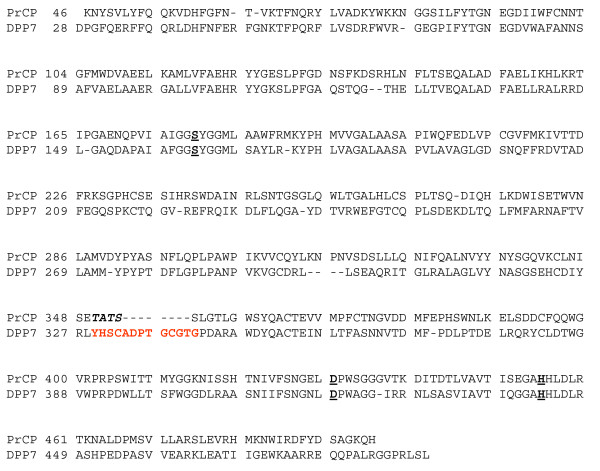
**Structure-based sequence alignment of DPP7 and PRCP**. Active site residues are marked in bold, underlined type. The DPP7-specific structural insertion is in red. The four disordered residues not modeled in the PRCP structure are italicized.

**Figure 7:**
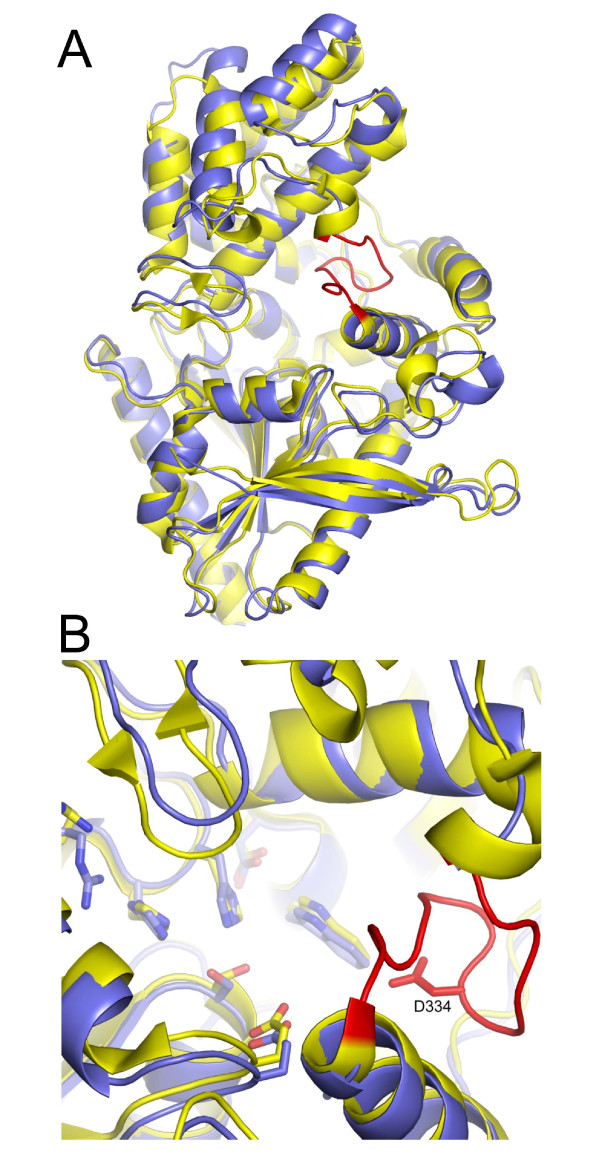
**Structural comparison of DPP7 and PRCP**. (A) Structural alignment of PRCP (blue) and DPP7 (yellow) and the sequence insertion of DPP7 that occludes the active-site cleft (red). (B) Enlarged view of site region with DPP7-specific insertion (red). Asp334 is shown as sticks.

PRCP shares 39.6% sequence identity with DPP7 (Figure 6). The crystal structure of DPP7 was recently deposited with the Protein Data Bank by the Structural Genomics Consortium (PDB code: 3JYH), although the structure is not yet described in print. Essentially all of the structural features observed for PRCP, including the α/β hydrolase domain, the novel SKS domain, the dimerization interface, the unusual Arg-His-His interaction in the active site, and the Pro S1 binding site are preserved between the two enzymes (Figures 7A-B). The C^α ^r.m.s.d. for the aligned structures is 1.20 Å. The structural description presented here for PRCP therefore defines the architecture of the S28 protease family fold.

**Figure 8:**
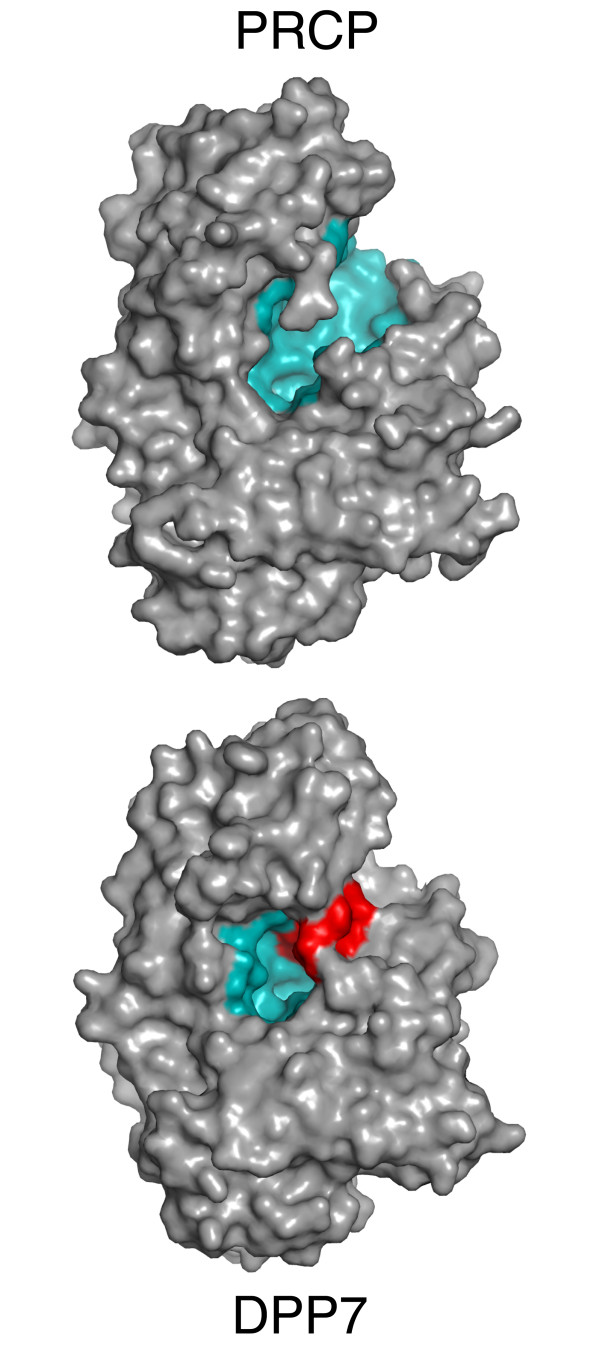
**The DPP7 substrate-binding groove is occluded by the DPP7-specific structural insertion**. Views of PRCP (top) and DPP7 (bottom) showing the elongated, solvent-accessible substrate binding groove of PRCP (teal), and the binding site of DPP7 (teal) that is truncated by the DPP7-specific insert (red).

A striking difference between PRCP and DPP7 is a structural insertion present only in DPP7 spanning residues 329-340 (Figures 6 and 7A). This short insertion sequence adopts a hairpin structure that is stabilized by a disulfide bridge between Cys 332 and Cys 338. The peptide substrates of DPP7 and PRCP must occupy the two enzymes in the same orientation based on the conserved architectures of the catalytic triad and the S1 Pro binding site. The structural insertion of DPP7 truncates the active-site cleft of DPP7 (Figure 8) and explains the different substrate specificities of the two enzymes. The binding of known substrates by PRCP that are cleaved at the C-terminus requires access to the long substrate binding groove of PRCP. In contrast, the DPP7-specific structural insertion creates a blocked substrate binding site that can only accommodate short, dipeptidyl extensions at the N-terminus of potential substrates. This represents a remarkably simple evolutionary adaptation to impart the C- and N-terminal substrate specificities of PRCP and DPP7 within a conserved active-site architecture.

The DPP7-specific insertion may also play an important role in substrate binding. For the aminopeptidase DPP4, substrate recognition involves coordination of the N-terminal amine of the substrate by Glu 206 of DPP4 (Figure 5B) [17,18]. The importance of this interaction in DPP4 is illustrated by the observations that the mutation of Glu 206 in DPP4 abolishes enzymatic activity [25] and that N-terminal acetylation of DPP4 substrates protects against DPP4 proteolysis [26]. The insertion loop of DPP7 also contains an acidic residue, Asp 334, which could function to coordinate with the N-terminus of the substrate in an analogous fashion to DPP4 (Figure 7B).

## Conclusions

The structure of the human carboxypeptidase PRCP presented here provides the first structural description of the S28 family of proteases. These proteases consist of a conserved α/β hydrolase domain and a novel structural domain that caps the active site. Comparison with the previously undescribed structure of the aminopeptidase DPP7 reveals that a short insertion sequence in DPP7 sterically occludes access to the substrate binding groove to provide a simple evolutionary adaptation to change substrate specificity. These structural results provide the basis for rational design of selective PRCP regulators for the modulation of cardiovascular and metabolic diseases.

## Methods

### Crystallization

Human PRCP was expressed, purified and crystallized as described previously [13]. Briefly, glycosylated PRCP was expressed as a secreted protein in CHO cells and purified using a combination of Ni-affinity, heparin and gel filtration chromatography. Crystals were obtained in 1.8 M ammonium sulfate, 0.1 M HEPES, pH 7.5, and 1-2% PEG 400 [13].

### Structure determination

The structure of PRCP was determined using MIRAS techniques (Table 1). Two heavy-atom derivatives were prepared by soaking native PRCP crystals in stabilizing solutions containing 5 mM ethyl mercurithiosalicylate or 2.5 mM K_2_PtCl_4 _for 2 or 10 days, respectively. Data were collected at the Advanced Light Source beamline 5.0.2 by Reciprocal Space Consulting. Diffraction images were integrated using XDS [27] and reduced using SCALA [28] as implemented in autoPROC (Global Phasing Limited, Cambridge, United Kingdom). Data sets were scaled together using SCALEIT [29], and heavy atom sites identified with SHELXD [30]. These heavy atom sites were used to seed runs of autoSHARP [31], combining native, mercury, and platinum data sets, to generate initial MIRAS phases and density-modified electron density maps. An initial model of PRCP was built into the 2.8 Å autoSHARP maps using Coot [32], and refined against the native data set at 2.8 Å using iterative rounds of autoBUSTER [33] refinement and manual rebuilding. MolProbity was used to evaluate the final refined model [34].

Figures were prepared with PyMOL [35]. Buried surface-area calculations were performed using AREAIMOL [29]. Structure alignments were performed using SSM [36] and DALI [37].

The PRCP coordinates have been deposited in the Protein Data Bank (PDB Code: 3N2Z)

## Abbreviations

DPP4: dipeptidyl peptidase 4; DPP7: dipeptidyl peptidase 7; FOM: figure of merit; MIRAS: multiple isomorphous replacement with anomalous scattering; PEP: prolylendopeptidase; PRCP: prolylcarboxypeptidase; r.m.s.d.: root mean square deviation.

## Authors' contributions

SMS, SBP, KWR, SS and KJL designed research. SMS, SBP, PDA, NJB, RED, DLH, REF, JCR and JMS performed research. SMS, SBP, KWR and KJL analyzed data. All authors read and approved the manuscript.
